# Extraction and quantification of biofilm bacteria: Method optimized for urinary catheters

**DOI:** 10.1038/s41598-018-26342-3

**Published:** 2018-05-23

**Authors:** Kedar Diwakar Mandakhalikar, Juwita Norasmara Rahmat, Edmund Chiong, Koon Gee Neoh, Liang Shen, Paul Anantharajah Tambyah

**Affiliations:** 1 Department of Medicine, National University of Singapore, Singapore, Singapore; 2 Department of Surgery, National University of Singapore, Singapore, Singapore; 3 Department of Chemical and Biomolecular Engineering, National University of Singapore, Singapore, Singapore; 4 Biostatistics Unit, National University of Singapore, Singapore, Singapore

## Abstract

Bacterial biofilms are responsible for the failure of many medical devices such as urinary catheters and are associated with many infectious and non-infectious complications. Preclinical and clinical evaluation of novel catheter coatings to prevent these infections needs to accurately quantify the bacterial load in the biofilm *in vitro* and *ex vivo*. There is currently no uniform gold standard for biofilm quantification for different surfaces and established biofilms. We have tried to establish a simple, accurate and reproducible method for extraction and measurement of biofilm bacteria on indwelling catheters, using a combination of vortexing and sonication. We demonstrate the usefulness of this method for catheters of different sizes – 3 Fr to 14 Fr – *in vitro*, in murine and porcine models, and indwelling in human clinical subjects. We also demonstrate consistent results with complex and polymicrobial biofilms. We believe that this standardized reproducible method will assist the assessment of biofilms in general and urological devices in particular in efforts to harness novel technologies to prevent healthcare associated infections.

## Introduction

### Bacterial biofilms and CAUTI

Biofilm formation is an important mechanism by which bacteria survive and persist despite antibiotics and host immune responses. Bacterial biofilms are responsible for the failure of many medical devices and are associated with many infectious and non-infectious complications^1^. Catheter associated urinary tract infections (CAUTI) are among the most commonly reported hospital acquired infections^2^. Bacteria frequently associated with CAUTI include uropathogenic *Escherichia coli* (UPEC), *Enterococcus spp*., *Klebsiella pneumoniae*, *Proteus mirabilis (P*. *mirabilis)*, *Pseudomonas aeruginosa*, etc.^3^. Intraluminal and extraluminal biofilms, caused by the attachment of bacteria to the catheter, lead to the entry and subsequent persistence of uropathogens in the bladder. In addition, urease producing bacteria, especially *P*. *mirabilis*, contribute significantly to catheter encrustation through precipitation of salts such as calcium and magnesium phosphates^4^. Together, these are the major contributors to morbidity and mortality in CAUTI. The role of bacterial biofilm and encrustation in CAUTI and its ensuing complications has been widely described in the literature^5–8^. Novel approaches to preventing CAUTI have focussed on preventing the development of biofilms using various techniques including novel coatings^9^. Thus, preclinical and clinical evaluation of novel catheter coatings or materials needs to accurately quantify the bacterial load in the biofilm to compare the effectiveness of these methods *in vitro* and *ex vivo*.

### Different ways to quantify biofilms reported in literature

Various methods have been used to analyse the bacterial population embedded in a biofilm. Semi quantitative methods such as rolling on agar plate or, quantification of viable or total cells after biofilm disruption by sonication (S), vortexing (V), swabbing, scrubbing, rinsing or scraping or using nitric oxide etc. have been widely described previously^10–13^. Comparisons of different methods have also been well reviewed^10–13^; however, there is no universally accepted gold standard for quantification of bacteria in biofilm. We summarize some of the studies using these techniques in Table 1. Short-time vortexing is widely used but it may not be enough to reduce the large cell clusters formed in older biofilms^14^. Sonication seems to be the more commonly used method for biofilm disruption from surfaces including catheters^15–18^.

**Table 1:** Summary of studies using different techniques for quantification of bacterial biofilms.

Method	Surface	Organism(s)	Time	Purpose
Biofilm dry weight measurement^27^	Acrylic slides	*S*. *epidermidis*	Up to 72 hr	To quantify differences in biofilm growth using batch and fed-batch culture methods
Impedance measurement^41^	24 well plate	*P*. *aeruginosa*	24 hr	To investigate the ability of impedance microsensors to detect biofilm presence using the impedance spectroscopy method.
Brief shaking and sonication in PBS^a^ supplemented with 0.05% Tween80^42^	24-gauge polyurethane IV catheter used as a urinary catheter	No inoculation but multiple spp. biofilms formed naturally	2–6 weeks	Establishing a rat model to mimic the actual clinical environment and to study biofilm development on urinary catheters
Real time PCR^b ^^43^	6-well polystyrene tissue culture plates	*S*. *aureus* clinical isolates	Up to 48 hr	To study transcriptional profiles of specific genes during biofilm production on polystyrene plates
Using nitrous oxide for biofilm dispersal^44^	Glass tubing, 96- well plate, petri dish with glass slide	*Pseudumonas aeruginosa*	6 days and 24 hr	To examine if reactive oxygen or nitrogen intermediates help in biofilm dispersal
Swabbing vigorously with sterile cotton swab^45^	Silicone-treated latex 8 Fr urethral catheters	No inoculation but multiple spp. biofilms formed naturally	Up to 5 days	To evaluate the efficacy and safety of a gentamicin sulphate coated urethral catheter
Fluorescence microscopy automatic counting system^14^	24 well plate	*S*. *epidermidis* strains	Up to 72 hr	To investigate the effects of sonication on the elimination of bacterial cell clusters from biofilms grown overtime
Sonication followed by vortex vs Roll plate method^11^	Central venous catheters	Not described	Mean 55 days (range, 4 to 469 days)	To compare the yields of 2 techniques to detect catheter tip colonization in patients with long-term tunnelled catheters
Sonication vs swabbing^10^	PVC^c^ slides	*Salmonella typhimurium* or *Pseudomonas fluorescens*	24 hr	To compare techniques for assessing biofilm populations

^a^Phosphate buffered saline.

^b^Polymerase chain reaction.

^c^Polyvinyl chloride.

### Necessity of a modified technique for urinary catheters

Many of the reported *in vitro* methods use flat surfaces to grow biofilms^19,20^. Curved surfaces and lumina of tubes present a different set of challenges in studying biofilm bacteria. Additionally, curved surfaces are also difficult to visualize under the microscope. Most of the studies on biofilm dislodging techniques, with some exceptions, describe biofilms that have grown only for 24–48 hrs. In clinical settings, where catheters remain *in situ* for days or weeks, biofilms develop over a longer period of time and are much sturdier and are more challenging to dislodge. Furthermore, crystalline biofilms responsible for CAUTI, in particular, are known to be often caused by multiple pathogens rather than single microorganisms^21^.

As part of the development of novel coated catheters^22^ we need to quantify biofilms from catheters recovered from experimental mice, micropigs, and also from human patients. Certain small animal experiments involve silicone tubing segments with a very small inner diameter (0.3–0.5 mm). The narrow lumen limits the techniques that can be used to disrupt the biofilm bacteria. A recent pilot study has demonstrated that combination of vortexing and sonication can enhance the bacterial yield from a biofilm grown *in vitro* on a PMMA coupon^19^. Holá *et al*. have previously used sonication-vortex-sonication to study microbial diversity in biofilms formed on urinary catheters^23^. However, extensive sonication is known to damage bacterial cells and thus it may affect viable colony counts. In view of these limitations, we tried to establish a simple method based on practical modifications of previously reported techniques to better document biofilms attached to urinary catheter surfaces – *in vitro* or *ex vivo*.

### Hypothesis and aim

We hypothesize that vortexing before sonication helps in dislodging any loosely attached layers of biofilm and enhances the effect of sonication on deep-seated layers that are strongly attached to the catheter surfaces. Further vortexing after limited sonication additionally helps in breaking down bacterial communities into individual cells and improves bacterial isolation thereby enabling a more accurate and uniform quantification without destroying bacterial cells excessively. More specifically, we performed this study to demonstrate that this is a consistent and reproducible method to extract and quantify microorganisms namely, *P*. *mirabilis* and *E*. *coli*, in crystalline as well as non-crystalline biofilms from urinary catheters recovered from mice, micropigs, and humans.

## Materials

RenaSIL-037–0.94 mm outer diameter (OD) – tubing (Braintree Scientific, Inc., USA) with or without surface modification was cut into 4–5 mm long segments and sterilised by autoclaving before use for mouse experiments. All-silicone Foley catheters [BardexVR, 14 Ch/Fr (OD 4.7 mm)] were purchased from C. R. Bard Inc., Georgia, and used for biofilm growth *in vitro*. Latex 2-way Foley catheters (14 Fr) were purchased from Urocare, Malaysia. Surface modification of these catheters was performed before using them for *in vivo* (micropig) experiments as previously described^22^.

*E*. *coli* UTI89, an uropathogenic strain known to be a good biofilm developer, isolated from a patient with uncomplicated cystitis, was kindly provided by Dr Swaine Chen of Genome Institute of Singapore^24^. *P*. *mirabilis* (ATCC 51286), a strain isolated from a patient with urinary catheter infection, was obtained from American Type Culture Collection (ATCC).

For growth in liquid media, *E*. *coli* was cultured in lysogeny broth (LB) and *P*. *mirabilis* was cultured in tryptic soy broth (TSB) (both from BD Difco). LB agar was used to grow *E*. *coli* on solid medium. Cysteine Lactose Electrolyte Deficient (CLED) agar was used for solid cultures of *P*. *mirabilis* to prevent swarming. Either CLED or a chromogenic medium - CHROMagar^TM^ – was used to grow unknown samples to help in preliminary identification.

C57BL/6 female mice were purchased from InVivos Pte Ltd., Singapore. Mice were used at the age of 18–20 weeks. PWG Micropigs® (3–4 years old) were purchased from Prestige BioResearch, Singapore.

Full length indwelling urinary catheters were also collected from human subjects with CAUTI, just after removal from the bladder as and when prescribed by clinicians.

## Methods

All mouse experiments were conducted in accordance with the university ethical regulations and were approved by the National University of Singapore – Institutional Animal Care and Use Committee (IACUC). All porcine experiments were conducted as approved by Prestige BioResearch Private Ltd, Singapore (PBR) – IACUC in accordance with the institutional guidelines. Work involving mice and pigs was funded by Technology Enterprise Commercialisation Scheme-Proof-Of-Concept grant of SPRING Singapore (TI/TECS/POC/14/10).

The study involving human subjects was approved by the local institutional research board NHG-DSRB and was funded by Singapore Ministry of Health (R-172-000368-511) and was undertaken in accordance with the NHG-DSRB guidelines and regulations. Informed consent was obtained from all the patients recruited in this study.

1. **Growing** ***E***. ***coli*** **UTI89 biofilm on urinary catheter segment** ***in vitro***
  i. Bacteria – A single bacterial colony was inoculated in 10 ml of LB broth and incubated overnight at 37 °C in static conditions. The bacteria were diluted and sub-cultured again overnight as 2 static serial passages have been shown to be essential for ideal type 1-pilus expression which plays a critical role in attachment of cells to catheter surface^25,26^.
    The passaged UTI89 culture was diluted in LB broth to ~5 × 10^5^ CFU/ml.
  ii. Catheter segment (mentioned as sample here onwards): Segments (1 cm long) of Foley catheter were immersed in 5 ml culture in a culture tube with sufficient aeration (in triplicates).
  iii. The biofilm was allowed to develop on samples by incubating at 37 °C (non-shaking) for 7 days using fed batch culture method i.e. growth medium replaced with fresh medium every 24 hours^27^.
2. **Biofilm growth** ***in vivo***
  i. Catheter samples from 3 mice with CAUTI were collected after 10–14 days of *E*. *coli* inoculation. Details of the mouse model of CAUTI are provided in the Supplementary material.
  ii. Full length indwelling catheters were collected after 25 days of catheter placement from 2 female micropigs (P1 and P2). Details of the porcine model of CAUTI are given in Supplementary material. These *ex vivo* catheters were cut into 1 cm segments from different regions of the catheter and biofilm bacteria were extracted to evaluate the efficacy of the method.
  iii. In addition to these animal models, full length indwelling urinary catheters were collected from 3 human patients recruited from a single centre in Singapore for an observational study designed to understand the genetics and epidemiology of UPEC in the female urinary tract.
3. **Extraction of biofilm**
  i. Washing the loosely attached planktonic bacterial cells from catheter segments.
    Full length catheters from micropigs and humans were cut into segments of 1 cm for ease of extraction and calculation. Mouse catheters were processed as 0.5 cm segments.
    - Samples were washed once by gently dipping in 5 ml sterile 1X PBS.
    - Remaining liquid from lumen was removed by gently tapping on a sterile absorbent paper (capillary action).
      Foley catheter segment has 2 lumens – large one for urine and small one for balloon inflation.
      Any leftover bacterial suspension in the lumen may influence the final counts.
  ii. Extraction of the biofilm using vortexing – sonication – vortexing (V-S-V) method.
    - Sample was transferred to 10 ml 1X PBS in a 50 ml tube.
    - Continuous vortex for 1 minute at full speed was performed.
    - Then, probe based sonication was conducted at 10 W (RMS) for 50–60 seconds [MICROSON^TM^ Ultrasonic Cell Disruptor XL-2000 using probe P-1 with a tip diameter of 3.2 mm].
      It may be necessary to optimize the strength and timing for a different sonicator.
      Keep the PBS with segments on ice to prevent heating due to sonication.
      Clean the probe with 70% ethanol and ddH_2_O between each sample.
    - Another round of continuous vortex for 1 minute at full speed was carried out.
  iii. To confirm that biofilm has been removed (almost) completely.
    - Samples were dip-washed once in sterile PBS and then transferred to 5 ml sterile LB broth.
    - The samples in LB broth were incubated at 37 °C with shaking at 200 rpm for 2 hrs and then serial dilutions were plated on nutrient agar.
      Doubling time for *E*. *coli* during steady state is ~20 minutes^28^. Even a small amount of contamination from the final PBS wash will grow to a stationary phase overnight. In our experience, 10^3^–10^4^ CFU/ml at the end of 2 hrs will grow even after the biofilm has been extracted. Therefore, we plated at the end of 2 hrs incubation.
4. **Plating**
  i. Sonicate was concentrated by centrifugation at 3100 g for 10 minutes. After carefully discarding 9 ml of supernatant, the pellet was resuspended in the remaining 1 ml PBS.
  ii. Ten-fold serial dilutions of the sonication supernatant were then prepared in PBS.
  iii. Serial dilutions including neat sonicate were plated on LB Agar plate by 20 µl drop (Miles and Misra) method in triplicate^29^.
    For porcine samples, CLED agar was used as it prevents swarming of *P*. *mirabilis*.
    For human samples, 20 μL of sonicate was also streak plated on CHROMagar^TM^ to identify other organisms.
    For this study, all serial dilutions were plated in triplicate and average CFU/cm was calculated.
  iv. Inverted agar plates were incubated at 37 °C (non-shaking) for 14–18 hours.
5. **Quantification of bacteria**
  i. After incubation, number of colonies per 20 µl drop was counted for each drop and the dilution was noted down. Average number of colonies was calculated.
    Count between 5 and 50 colonies per drop.
  ii. ‘CFU per cm’ of a segment was calculated as final quantification using the formula: CFU/cm = Average number of colonies for a dilution × 50 × dilution factor.
    The formula is adjusted according to the length of the catheter segment.
6. **Scanning electron microscopy (SEM)** was performed as published elsewhere^30^ with some modifications
  i. The samples were fixed in 2.5% glutaraldehyde in PBS at 4 °C overnight.
  ii. After three washes with PBS, samples were post-fixed with 1% OsO_4_ reagent for 1–2 hours.
  iii. Next action was step-wise dehydration in increasing percentages of ethanol from 25% up to 100%.
  iv. The dehydrated samples were then dried with CO_2_ in a critical point drier.
  v. The samples were mounted on stubs and sputter coated with a thin layer of gold.
  vi. The coated samples were visualized using a JEOL JSM-6701F Field Emission Scanning Electron Microscope.
7. **Statistics**

Extraction of biofilm grown *in vitro* was performed with V-S-V method and compared with 5 other meth-ods – V, S, Vortexing-Vortexing (V-V), Sonication-Vortexing (S-V) and Vortexing-Sonication (V-S). This *in vitro* experiment was performed four times with three replicates for each method in each experiment. Consecutive 1 cm segments from 3 different regions of the catheters recovered from human patients were processed using S-V, V-V, and V-S-V methods for comparison. Mixed model^31^ was utilized to compare the results of different methods and the variance and covariance matrix was modelled by compound symmetric structure. Location was adjusted (for human catheter samples), and nature logarithm transformation was applied to bacterial counts before mixed model was carried out due to normality assumption.

## Results and Discussion

### Results – Comparison between different methods

**Figure 1:**
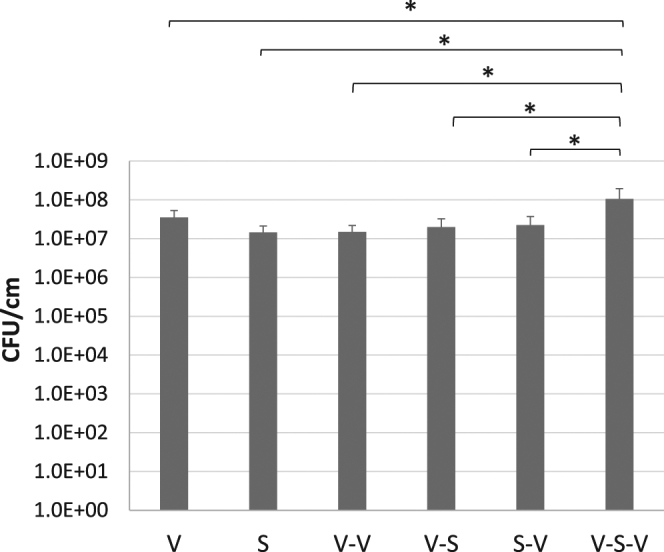
Comparison of biofilm extraction by different methods *in vitro*. Average extraction of *E*. *coli* biofilm grown *in vitro* on 1 cm segments of silicone catheter for 7 days. V and S indicate vortexing and sonication respectively. Y-axis is in log_10_ scale. Error bars indicate *SD*. *indicates *P* < 0.005 with mixed model analysis.

A comparison of the efficiency of different combinations of vortexing and sonication in extraction of bacteria forming biofilm is demonstrated in Fig. 1, which shows average of extraction of *E*. *coli* biofilm grown *in vitro* on 1 cm segments of silicone catheter for 7 days (12 replicates per method). V-S-V method yielded 3 to 7 times more bacteria from biofilm as compared to V, S, V-V, V-S and S-V. The difference was statistically significant as mixed model analysis showed *P* value to be lower than 0.005 for each of the five methods as compared to V-S-V.

**Figure 2:**
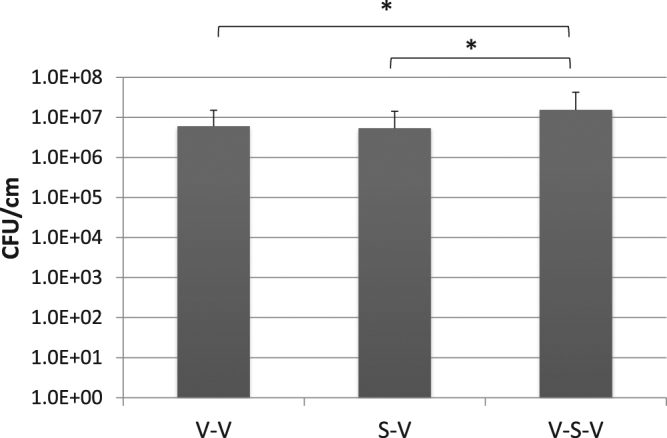
Comparison of biofilm extraction by different methods *ex vivo*. Average CFU/cm extracted from consecutive segments from 3 different regions of a urinary catheter collected from 3 human patients. V and S indicate vortexing and sonication respectively. Y-axis is in log_10_ scale. Error bars indicate *SD*. *indicates *P* < 0.005 with mixed model analysis.

These *in vitro* results were confirmed using consecutive 1 cm segments from 3 different regions of indwelling urinary catheters collected from 3 human patients. The average CFU values of the biofilm extracted from 1 cm segments from different regions of the catheter are shown in Fig. 2. It can be observed that V-S-V method extracts 2.6 to 2.9 times more bacteria from biofilm than other combinations of these techniques. Furthermore, mixed model analysis shows that there is significant difference among 3 methods, and V-S-V gave significantly higher CFU as compared to V-V (*P* < 0.005) and S-V (*P* < 0.005) methods.

### Results – *ex vivo*

We confirmed the reproducibility of the method for catheters by quantification of bacteria extracted from biofilms on 3 Fr (0.64 mm inner diameter) urinary catheters recovered from mice. One catheter segment was collected post mortem from each mouse and subjected to biofilm extraction via V-S-V method. Consistent and high values of >10^8^ CFU/cm were obtained from the catheter segments (see Supplementary Fig. S1). The results indicate that the method is consistent and works well with small lumen catheters with non-crystalline biofilms.

**Figure 3:**
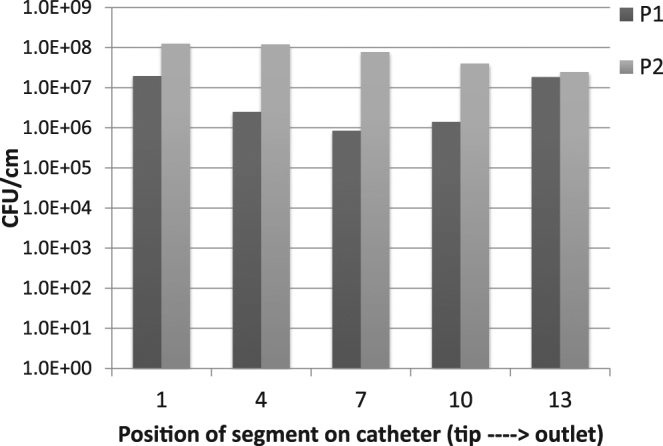
Representative quantification of bacterial biofilm extracted from urinary catheters recovered from porcine CAUTI model. Urinary catheters were indwelling in micropigs P1 and P2 for 23 and 25 respectively. Five segments (1 cm) along the length of the catheters were subjected to V-S-V biofilm extraction. P1 was uninoculated whereas P2 was intravesically inoculated with *P*. *mirabilis* on the day of catheterization. Y-axis is in log_10_ scale.

Colony counts from 1 cm segments (CFU/cm) from 5 different sections of urinary catheters recovered from micropigs P1 and P2 after 23 and 25 days respectively are shown in Fig. 3. X-axis indicates the position of the segment on the catheter – 1 being the tip and 13 being the region where catheter exits the body of the animal. With our method, we could successfully extract crystalline biofilms with high yields up to >10^8^ CFU/cm. Furthermore, bacteria belonging to multiple species were isolated from P2 which indicates that this method works well with polymicrobial biofilms. However, due to the limited number of samples from animal models, and based on our *in vitro* results we used only the V-S-V method for the *ex vivo* samples.

Multiple species of bacteria were also isolated from catheters recovered from human patients. As shown in Supplementary Fig. S2 – a representative agar plate – at least 3 different species can be identified preliminarily in pink, metallic blue and turquoise blue colonies. Each species can then be identified by standard methods and quantified.

### Correlation

**Figure 4:**
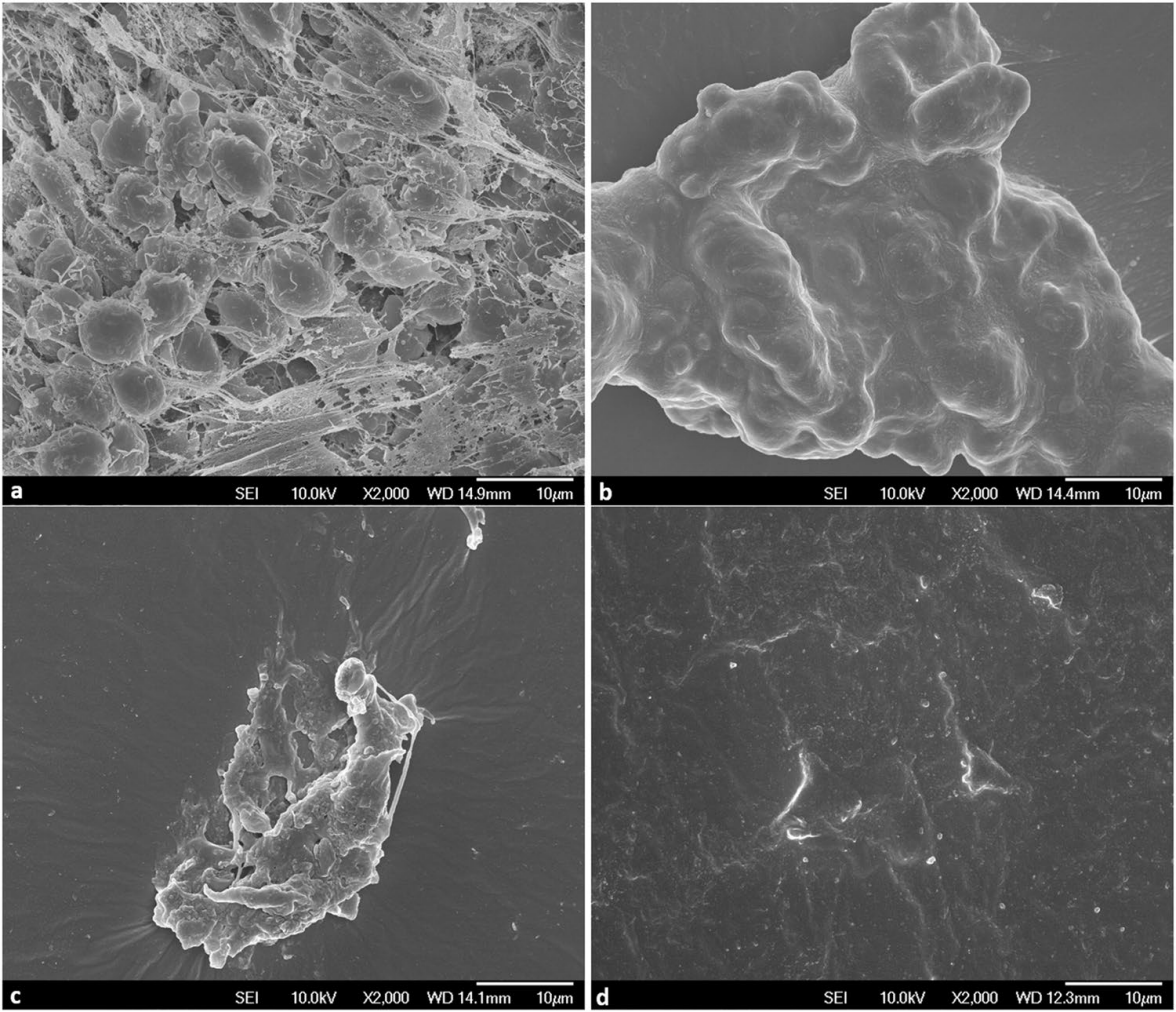
Representative scanning electron microscopy images showing differences in biofilm removal by different methods. Indwelling urinary catheter was collected from human patient and consecutive 5 mm segments were used for analysis. Images were scanned from 3 random areas on the surface. (**a**) Untreated control, (**b**) V-V, (**c**) S-V, (**d**) V-S-V.

The quantification results were also validated with visualization with electron microscopy. Observable differences in the biofilm extraction from human samples by V-S-V and the other combinations are demonstrated in Fig. 4. It can be seen that there is comparatively less residue after biofilm bacteria are extracted with V-S-V. Images were scanned randomly from 3 areas of the surface of catheter segments and representative images are shown here.

Crystal violet (CV) staining followed by destaining with ethanol and quantifying the optical absorbance measurements of reclaimed stain is another indirect method for biofilm quantification^1^. We also attempted to validate the V-S-V method using this assay but were unsuccessful in getting consistent readings from curved surfaces and small lumina. We observed high background retention of stain by the catheter segments due to surface tension in the smaller lumen as shown in Supplementary Fig. S3.

## Discussion

In this study, we have tried to establish a simple method for accurate and reproducible extraction and measurement of biofilm forming bacteria that can be used for various urinary catheters in investigating novel ways for prevention of CAUTI. Using this method, we have successfully isolated and quantified bacteria from biofilms growing *in vitro*, and on urinary catheters recovered from experimental mice and micropigs, as well as human patients. Additionally, this method also works with crystalline as well as non-crystalline biofilms as seen with *P*. *mirabilis* inoculation in micropigs and UPEC inoculation in mice. We have also shown that this method performs well for different sizes of catheters; we have used 3 Fr for implantation in mice and 14 Fr for micropigs and human patients. This suggests that the method can be used in a variety of experimental settings.

Our results are consistent with a previous study that reports that individually, sonication and vortexing, were capable of removing only the top layers of bacterial cells in a biofilm but not the EPS residues or the cells attached to the surface^32^.

Analysis of biofilms on surfaces using techniques such as sonication depends on the detachment of the bacterial cells from the surfaces and subsequent enumeration of colony forming units by standard microbiological techniques. These methods help to directly quantify the bacterial load in the biofilm. However, the structure and physiology of the bacterial biofilm cannot be evaluated with overly destructive methods^33^. Furthermore, within a biofilm, bacteria are known to be at different stages of growth and metabolism which also means that only the viable and cultivable bacterial cells detached by sonication that grow on nutrient media can be quantified^34^. This is a major limitation of this method which we have attempted to deal with by limiting the sonication to a minimum in between two vortexing stages.

The duration of sonication may also influence the cell structure and / or metabolism of bacteria and it has been reported that sonication more than 5 minutes can be harmful for the cell viability^35^. The destructive techniques could also be harsh on biofilm forming bacteria which may result in viable but non-culturable bacteria. It has to be noted that Gram negative bacteria are more sensitive to sonication than Gram positive bacteria^36^. We also tested the effect of sonication on bacterial suspension by plating before and after sonication (see Supplementary Fig. S4) and found that the sonication strength and duration that we used did not considerably damage (12–14%) our Gram-negative bacteria (*E*. *coli* and *P*. *mirabilis*) which are the major uropathogens. Another factor that influences the outcome considerably is type of sonicator, whether a probe-based ultrasonic processor or an ultrasonic water bath. It has to be noted that even though both the techniques are ultrasound-based, the intensity of cavitation in water-bath sonicator is low and the effect can be uneven^37^. Hence, we have used a probe based sonicator for extraction of well-established bacterial biofilms, which seems to have worked well. However, it may be necessary to optimize the strength and duration of sonication to suit a different sonicator (whether probe or water bath).

Non-destructive methods to study biofilm typically involve imaging – microscopy with phase contrast or bacteria-specific staining. We have successfully used SEM to observe bacterial biofilms. While SEM is useful to qualitatively assess biofilms, quantitation and species identification would require culture-based techniques. CV assay can be useful when comparing between two or more similar samples. Limitations of this technique are the need for a non-absorbent surface for biofilm growth, a standard staining and destaining time, and a standard wavelength to read absorbance as well as its inability to identify polymicrobial biofilms^1^. Published studies using colorimetric methods have been done on mostly on flat surfaces and we are unable to find many publications reporting *in situ* biofilm quantifications from curved surfaces^38–40^. To the extent of our knowledge, CV assay has not been used for biofilm quantification *ex vivo*.

Thus, in absence of a gold standard, our method can provide a simple, accurate and reproducible method for extraction and measurement along with identification of biofilm bacteria and will help in the assessment of biofilms in general and urological devices in particular in efforts to harness novel technologies to prevent healthcare associated infections.

## Conclusion

Bacterial biofilms associated with medical devices are responsible for a number of diseases. In preclinical and clinical evaluation of novel anti-microbial coatings or materials for these medical devices, it is of great importance to precisely quantify the bacterial load in the biofilm. As there is no established gold standard method to quantify bacterial biofilms on medical devices, in this study, we investigated the combination of two simple methods to establish a consistent method to extract and quantify crystalline as well as non-crystalline biofilms (<10^6^ to >10^8^ CFU/cm) from catheters of different sizes. We believe that the V-S-V method is an efficient means for extraction of biofilms on medical devices for accurate assessment of the various important interventions to prevent device associated infections, especially, those in patients with long term catheterization.

## Electronic supplementary material

Supporting information
