# Supplementary material for: Extraction and quantification of biofilm bacteria: Method optimized for urinary catheters
Source: Sci Rep. 2018 May 23;8:8069. doi: 10.1038/s41598-018-26342-3 (PMC5966383; doi:10.1038/s41598-018-26342-3)
Supplement: Supplementary file 1 — Supporting information [file 41598_2018_26342_MOESM1_ESM.pdf]

Supporting information

Extraction and quantification of biofilm bacteria: Method optimized for urinary catheters

Kedar Diwakar Mandakhalikar, Juwita Norasmara Bte Rahmat, Edmund Chiong, Koon Gee Neoh, Liang Shen, Paul Anantharajah Tambyah

Materials and methods:

Biofilm growth *in vivo*

1) Mouse model of CAUTI – Catheter segments (RenaSil 037 – 5 mm long) were transurethrally placed in urinary bladders of C57BL/6 mice as described previously by Kadurugamuwa *et al.* [1] and kept indwelling for up to 14 days. Catheter segments were indwelling in mouse M1 for 10 days and in mice M2 and M3 for 14 days with intravesical inoculation of uropathogenic *E. coli* - UTI89 strain.

2) Porcine model of CAUTI – Similarly, 14 Fr Foley catheters (with a novel silver antibacterial coating [2]) were transurethrally placed in female micropigs (P1 and P2) and kept indwelling for up to 25 days. P2 was intravesically inoculated with *P. mirabilis* on the day of catheterization. Catheters were collected from the pigs at the end of the experiment and cut into 1 cm segments. Five segments along the length of the catheters were subjected to V-S-V biofilm extraction.

Results:

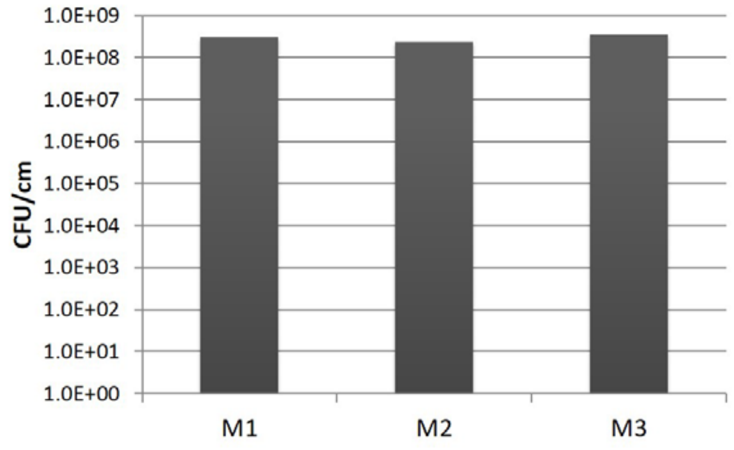

Supplementary Figure S1 Quantification of bacterial biofilm extracted from urinary catheters recovered from mouse model. One urinary catheter each was indwelling in mice M1, M2 and M3 for 10-14 days. Mice were inoculated with *E. coli* on the day of catheterization. Y-axis is in $\log_{10}$ scale.

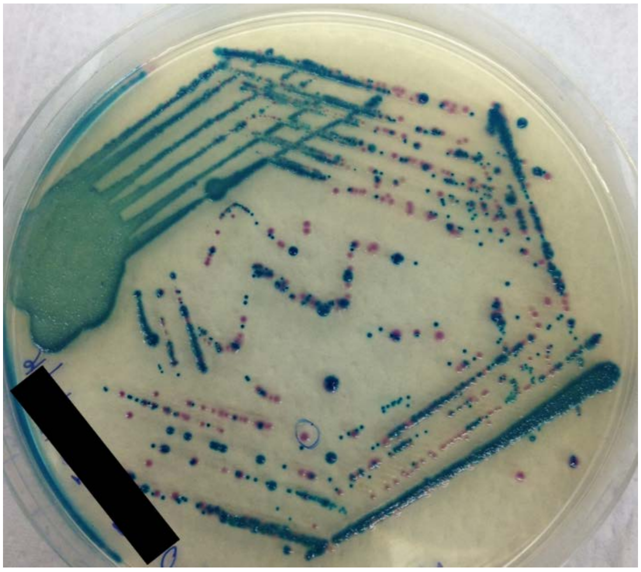

Supplementary Figure S2 Isolation of bacteria from multi species biofilm extracted from the tip of urinary catheter recovered from a human patient.

Correlation:

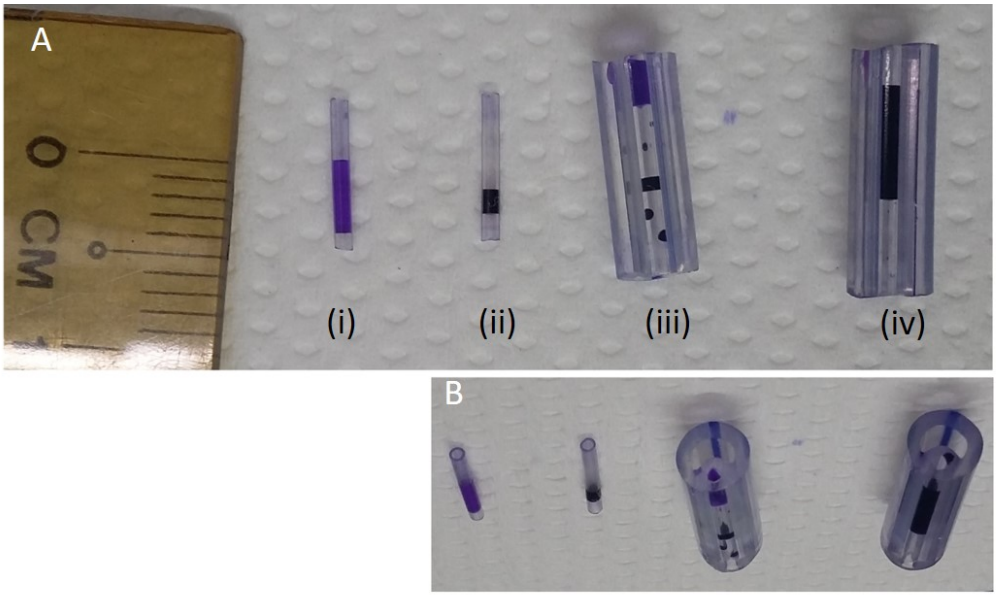

Supplementary Figure S3 Background retention of stain. Blank catheters without biofilm also showed high values due to excess stain persisting in the small lumen of the catheter segments leading to inconsistent results.
Panel A (i) retention of destaining solution and (ii) CV stain in mouse catheters (iii) retention of destaining solution and CV stain and (iv) CV stain in human catheter segments. Panel B shows the same segments at a tilted angle to show that stain is retained inside the small lumen and not inside the larger lumen of human catheter.

Discussion:

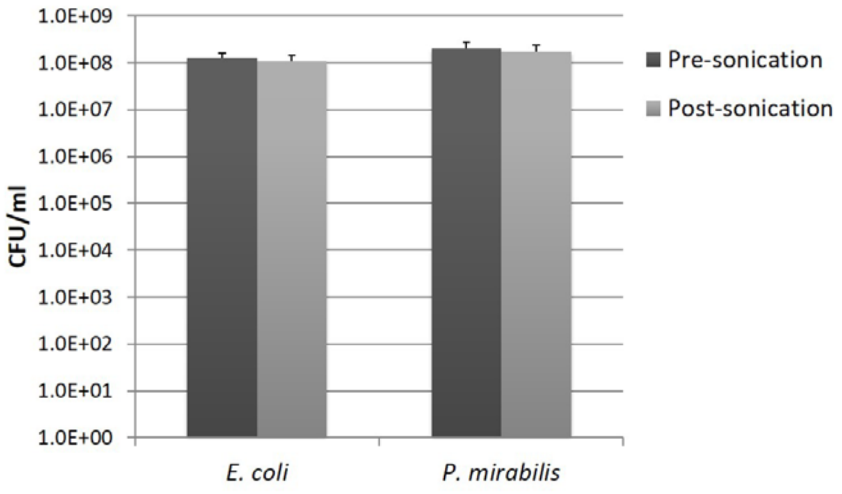

Supplementary Figure S4 Average quantification of bacteria pre- and post-sonication. Samples were randomly selected on 3 different days during optimization of the method and plated before and after sonication for 1 minute. Error bars indicate *SD*. Y-axis is in $\log_{10}$ scale.

References

1 Kadurugamuwa, J. L. *et al.* Noninvasive biophotonic imaging for monitoring of catheter-associated urinary tract infections and therapy in mice. *Infect Immun* **73**, 3878-3887, doi:10.1128/IAI.73.7.3878-3887.2005 (2005).

2 Wang, R., Neoh, K. G., Kang, E. T., Tambyah, P. A. & Chiong, E. Antifouling coating with controllable and sustained silver release for long-term inhibition of infection and encrustation in urinary catheters. *J Biomed Mater Res B Appl Biomater* **103**, 519-528, doi:10.1002/jbm.b.33230 (2015).
